# The use of metabolic profiling to identify insulin resistance in veal calves

**DOI:** 10.1371/journal.pone.0179612

**Published:** 2017-06-15

**Authors:** Andre J. Pantophlet, Han Roelofsen, Marcel P. de Vries, Walter J. J. Gerrits, Joost J. G. C. van den Borne, Roel J. Vonk

**Affiliations:** 1 Department of Pediatrics; Center for Liver, Digestive and Metabolic Diseases, University Groningen, University Medical Centre Groningen, Groningen, The Netherlands; 2 Medical Biomics, University Groningen, University Medical Centre Groningen, Groningen, The Netherlands; 3 Animal Nutrition Group, Wageningen University, Wageningen, The Netherlands; Medical University of Vienna, AUSTRIA

## Abstract

Heavy veal calves (4–6 months old) are at risk of developing insulin resistance and disturbed glucose homeostasis. Prolonged insulin resistance could lead to metabolic disorders and impaired growth performance. Recently, we discovered that heavy Holstein-Friesian calves raised on a high-lactose or high-fat diet did not differ in insulin sensitivity, that insulin sensitivity was low and 50% of the calves could be considered insulin resistant. Understanding the patho-physiological mechanisms underlying insulin resistance and discovering biomarkers for early diagnosis would be useful for developing prevention strategies. Therefore, we explored plasma metabolic profiling techniques to build models and discover potential biomarkers and pathways that can distinguish between insulin resistant and moderately insulin sensitive veal calves. The calves (n = 14) were classified as insulin resistant (IR) or moderately insulin sensitive (MIS) based on results from a euglycemic-hyperinsulinemic clamp, using a cut-off value (M/I-value <4.4) to identify insulin resistance. Metabolic profiles of fasting plasma samples were analyzed using reversed phase (RP) and hydrophilic interaction (HILIC) liquid chromatography–mass spectrometry (LC-MS). Orthogonal partial least square discriminant analysis was performed to compare metabolic profiles. Insulin sensitivity was on average 2.3x higher (*P* <0.001) in MIS than IR group. For both RP-LC-MS and HILIC-LC-MS satisfactory models were build (R^2^Y >90% and Q^2^Y >66%), which allowed discrimination between MIS and IR calves. A total of 7 and 20 metabolic features (for RP-LC-MS and HILIC-LC-MS respectively) were most responsible for group separation. Of these, 7 metabolites could putatively be identified that differed (*P* <0.05) between groups (potential biomarkers). Pathway analysis indicated disturbances in glycerophospholipid and sphingolipid metabolism, the glycine, serine and threonine metabolism, and primary bile acid biosynthesis. These results demonstrate that plasma metabolic profiling can be used to identify insulin resistance in veal calves and can lead to underlying mechanisms.

## Introduction

Veal calves are fed milk replacer (**MR**), roughage and concentrates. A large portion (60–70%) of the digestible nutrient intake originates from MR. The MR contains large amounts of lactose and fat, approximately 45% lactose and 20% fat on DM basis. Persistently high intakes of lactose and fat may lead to dysregulations in glucose homeostasis, which are characterized by a high incidence of hyperglycemia, hyperinsulinemia and glycosuria. These problems have been identified in heavy (4–6 months old) veal calves [1–3]. In addition, a substantial decrease in insulin sensitivity is observed in calves during the first months of life [4, 5]. In a recent study with heavy veal calves raised on a high-fat or high-lactose MR diet we observed that insulin sensitivity values were low (averaging 4.2 ± 0.5 x 10^−2^ [(mg/(kg*min))/(μU/mL)]), and 50% of the calves develop insulin resistance (when comparing insulin sensitivity values with human cut-off values for defining insulin resistance; [6]). In order to prevent the development of insulin resistance, it is of importance to understand the patho-physiological mechanisms of insulin resistance and to identify early biomarkers of decreased insulin sensitivity. By detecting decreased insulin sensitivity at an early stage, management and feeding strategies could be developed to prevent the development insulin resistance. Therefore, we investigated the applicability of metabolomic profiling techniques to identify insulin resistance in veal calves.

Metabolomics focuses on the analysis of the metabolome together with pattern recognition techniques to highlight and monitor metabolic changes related to disease status or nutritional intervention [7, 8]. Its potential has been demonstrated in the diagnosis of several metabolic diseases [9–11]. In the current exploratory study we applied metabolic profiling, to build models to discover potential biomarkers and pathways related to insulin resistance in veal calves.

## Material and methods

### Animals and housing

Sixteen male Holstein-Friesian calves (120 ± 2.8 kg BW; 99 ± 2.0 d old) were purchased and housed at the experimental facilities of Wageningen University. During the first 6 weeks of the 13-week study, calves were housed in pens of 4 calves each (2 m^2^ per calf), which were fitted with a wooden slatted floor and galvanized fencings. Then, calves were transferred to metabolic cages (dimension: 0.80 x 1.8 m) for the next 7 weeks, during which whole-body insulin sensitivity was measured (see experimental procedures). Ventilation occurred by ceiling fans, and illumination by natural light and artificial (fluorescent lamps) light between 0700 and 1900 h. Temperature and humidity were controlled at 18°C and 65% respectively.

The study was conducted in 2011. Experimental procedures complied with the Dutch Law on Experimental Animals, and the ETS123 (Council of Europe 1985 and the 86/609/EEC Directive) and were approved by the Animal Care and Use Committee of Wageningen University.

### Experimental design, diets and feeding

A detailed description of the experimental design, diets and feeding were described previously [6]. Briefly, calves were assigned to either a high-lactose diet (HL; n = 8) or a high-fat diet (HF; n = 8), and to 1 of 8 blocks (pairs of calves) with one HL calf and one HF calf per block. Due to health problems in two HF calves, block seven consisted of two HL calves and block 8 (with the two remaining HF calves) was not included in the whole-body insulin sensitivity and metabolomic profiling measurements. Lactose and fat were exchanged iso-energetically between treatments based on digestible energy. MR was fed on individual basis twice a day (0800 and 1630 h). In addition, solid feed was provided per pen when calves were housed in groups and per individual calf when housed separately on metabolic cages. Solid feed was provided once a day. Calves had *ad libitum* access to drinking water throughout the study. At end of the study, calves were euthanized by an intravenous injection of sodium pentobarbital.

### Experimental procedures

A detailed description of the experimental procedures were given elsewhere [6]. In short, whole-body insulin sensitivity was assessed by the euglycemic-hyperinsulinemic clamp technique in seven consecutive weeks (i.e. experimental week 7–13; 1 block per week). Semi-permanent catheters (Careflow, Becton Dickinson, Alphen aan den Rijn, The Netherlands) were inserted in both jugular veins. Calves were fasted for 15 h (morning feed omitted) to achieve a steady glucose turnover rate prior to the measurements. Before starting the 4-h clamp study, three 5 mL blood samples were taken from -40 to -10 min (before infusion) to determine basal plasma glucose concentrations. At start of the clamp, a priming dose of insulin of 2.1 mU/kg BW/min (Actrapid 100 IE/mL, Novo Nordisk, Denmark) was infused into the left jugular vein catheter, within 5 min, to rapidly increase the plasma insulin concentration. Then, the rate of insulin infusion was decreased and maintained at 1 mU/kg BW/min for a period of 4 h (plasma insulin levels ~135 mU/L). At t = 5 min glucose (20% glucose solution for intravenous infusion; B. Braun, Melsungen, Germany) was continuously infused to maintain basal plasma glucose concentration, hence the infusion rate was adjusted to the glucose clearance rate.

During the clamp study, 0.3 mL blood samples were taken from the catheter in 10-min and 15-min intervals during 0–2 hours and 2–4 hours respectively. In these samples, plasma glucose concentrations were measured using Precision Xtra Plus test strips in combination with the Precision Exceed Sensor (Abbott, Weesp, The Netherlands).

In addition, 5 mL blood samples were taken in 30-min intervals for the analysis of plasma glucose and insulin concentrations. Blood was collected in sodium fluoride vacutainer tubes for glucose and in heparin vacutainer tubes for insulin (BD diagnostics, Breda, The Netherlands). Samples were centrifuged (1,500 x *g* for 12 min) and plasma was harvested and stored at -20°C until analysis.

Plasma glucose was analyzed on an Architect ci8200 analyzer using the hexokinase method (Abbott Laboratories, Chicago, IL, USA) and plasma insulin was analyzed using a Coat-a-Count radioimmunoassay kit (Siemens Healthcare Diagnostics, Erlangen, Germany). The within- and between-run coefficients of variation for glucose were ≤2%. The within- and between-run coefficients of variation for insulin were ≤5% and ≤7%, respectively.

The glucose infusion rate (**GIR**) was adjusted (depending on the changes in plasma glucose level) to maintain a constant, basal plasma glucose level during insulin infusion. Glucose disposal (**M-value**) was defined as the average GIR at steady state divided by BW. Whole-body insulin sensitivity was defined as the M-value divided by the average plasma insulin level at steady state (**M/I-value**).

### Metabolomic profiling

Plasma metabolic profiling was performed using reversed phase (**RP**) and hydrophilic interaction (**HILIC**) liquid chromatography–mass spectrometry (**LC-MS**). These techniques are complementary, with RP-LC-MS able to separate and detect nonpolar to weakly polar metabolites and HILIC-LC-MS able to separate and detect weakly polar to polar metabolites. LC-MS was performed using a UFLC Prominence system (Shimadzu, Kyoto, Japan) coupled to a high-resolution LTQ-Orbitrap XL mass spectrometer (Thermo Fisher Scientific, Bremen, Germany), equipped with an Ion Max electrospray source. Analyses were performed in both positive and negative ionization mode. Mass spectrometric data was acquired in the centroid mode over the range of 100–800 m/z at a resolution of 60,000 at m/z 400. Low-resolution collision induced dissociation fragmentation data using the LTQ (MS/MS) was also acquired to facilitate compound identification in a TOP-n data dependent acquisition.

#### Sample preparation

Plasma samples were allowed to thaw at 4°C for 6 hours. Then, 800 μL of a methanol/acetonitrile/acetone (1:1:1 v/v) solution was added to 200 μL plasma each study sample. The mixture was gently vortexed at 4°C for 15 min and centrifuged at 12,500 x g for 10 min at 4°C. Then, 800 μL of the supernatant was evaporated to dryness under a gentle stream of nitrogen at 30°C. The residue was reconstituted in 100 μL methanol and 300 μL elution solvent A (see below for composition) for RP and in 400 μL elution solvent B (see below for composition) for HILIC. In addition, a quality control (**QC**) sample and a blank sample were prepared. The QC sample was prepared by mixing 100 μL of each study sample (to represent the biochemical diversity of the study samples), and processed identical to the study samples. For the blank sample Milli-Q water was used. Sample processing was identical to the study samples.

#### Sequence of injection

The analytical run started with the blank sample (injected 3 times for background subtraction), followed by the QC sample (injected 6 times for column conditioning; not used for data analysis). Then, the study samples were injected in random order. The QC sample was injected again after every 3 study samples (and at the end of the run) to calculate the analytical precision for each metabolic feature.

#### Reversed-phase chromatography

For reversed-phase chromatography a Kinetex C18 column (100 mm × 2.1 mm, 2.6 μm particles) with a SecurityGuard column (2.1 mm × 2 mm, 2 μm particles) was used (Phenomenex, Torrance, CA, USA). The column temperature was set at 35°C and the autosampler temperature was 5°C. The gradient elution solvents were A; 95:5 water-acetonitrile (v/v), containing 5 mM ammonium formate and 0.1% formic acid (v/v), and B; 95:5 acetonitrile-water, containing 5 mM ammonium formate and 0.1% formic acid. The gradient (A:B, v/v) was as follows: an isobaric period at 98:2 for 5 min, followed by a linear gradient from 98:2 to 2:98 in 25 min, then held at 2:98 for 5 min, followed by a linear gradient change from 2:98 to 98:2 in 1 min, then held at 98:2 for 5 min. The flow rate was 0.2 mL/min.

#### HILIC chromatography

For HILIC chromatography a Kinetex HILIC column (100 mm × 2.1 mm, 2.6 μm particles) with a SecurityGuard column (2.1 mm × 2 mm, 2 μm particles) was used (Phenomenex, Torrance, CA, USA). The column temperature was set at 35°C and the autosampler temperature was 5°C. The gradient elution solvents were A; 95:5 water-acetonitrile (v/v), containing 5 mM ammonium formate and 0.1% formic acid (v/v), and B; 95:5 acetonitrile-water, containing 5 mM ammonium formate and 0.1% formic acid. The gradient (A:B, v/v) was as follows: an isobaric period at 5:95 for 5 min, followed by a linear gradient from 5:95 to 50:50 in 25 min, then held at 50:50 for 5 min, followed by a linear gradient change from 50:50 to 5:95 in 1 min, then held at 5:95 for 5 min. The flow rate was 0.2 mL/min.

#### Mass spectrometry

The electrospray MS settings for both RP and HILIC were as followed: spray voltage 4.5 kV for positive ionization mode (3 kV for negative mode) and the capillary temperature was set at 250°C for positive ionization mode (250°C for negative mode). Nitrogen sheath gas and auxiliary gas were set at 25 and 15 arbitrary units, respectively.

### Data processing and statistical analysis

The raw LC-MS data were processed with Sieve 2.2 (Thermo Scientific) using the default settings except for the minimal signal to noise ratio for peak detection, which was set at 5. Metabolomic features with a coefficient of variation (of the normalized peak area) of the QC samples >25% were excluded from the dataset. The spectral data was then exported to Excel and results of the positive and negative mode analyzed with the same technique (i.e. RP or HILIC) were merged. After data processing, a multivariate analysis was conducted using SIMCA-P (Umetrics, Sweden). The data was pareto-scaled and subjected to orthogonal projection to latent structures discriminant analysis (OPLS-DA). The quality and reliability of the models were assessed by R^2^Y, representing the explained variation described by the model, and Q^2^Y, representing the predictive power of the model (based on the default 7-round cross validation procedure used in SIMCA-P). Permutation tests (n = 100) were performed to assess the robustness of the models. Also, a CV-ANOVA was calculated to assess the reliability of the models.

The variable importance in the projection (VIP) was used to identify the metabolic features that most significantly contributed to the clustering of groups within the OPLS-DA models [12]. Metabolic features with a VIP ≥2.0 were considered important. Also, an independent t-test was performed (using SPSS version 22, IBM, SPSS Inc., Chicago, IL) on all metabolic features with a VIP ≥2.0 to highlight which of these metabolic features also differ at univariate level between groups. A *P*-value of ≤0.05 was considered significant. Metabolic features with VIP ≥2.0 and *P* ≤0.05 were considered potential biomarkers.

### Metabolite identification and pathway analysis

Metabolite identification was performed on the potential biomarkers, and was achieved by comparing spectral data (exact mass and MS/MS) with data available from the human metabolome database (http://hmdb.ca/) and the METLIN database (http://metlin.scripps.edu/). When available, in-house, putative ID’s were confirmed by comparison with authentic standards (retention time, exact mass and MS/MS).

Pathway analysis was performed on the (putatively) identified biomarkers to highlight pathways that were disturbed due to the reduced insulin sensitivity. Metaboanalyst (version 2.0), a web-based program that uses the KEGG (http://www.genome.jp/kegg/) pathway database [13] was used for analysis. The *Bos Taurus* library was chosen for pathway analysis.

## Results

### Insulin sensitivity

Insulin sensitivity ranged from 1.5 to 8.3 x 10^−2^ (mg/(kg*min))/(μU/mL) between calves and was not differentially affected by dietary treatment (*P* >0.05; [6]). 50% of all calves had an insulin sensitivity (M/I-value) <4.4 and thus were considered insulin resistant (**IR**). Other calves were classified as moderately insulin sensitive (**MIS**). Whole- body insulin sensitivity differed substantially (*P* <0.001) between IR and MIS calves (Table 1) and ranged from 2.1–3.8 in IR calves and 4.4–8.2 in MIS calves.

**Table 1:** Characteristics of insulin resistant (IR) vs. moderately insulin sensitive (MIS) veal calves.

	IR	MIS	*P*-value^4^
High-Fat diet, n	4	2	-
High-Lactose diet, n	3	5	-
Age, days	169±10	165±15	0.750
BW, kg	248±7	241±6	0.483
Insulin^1^ (mU/L)	135±5	133±3	0.640
M-value^2^ (mg/BW/min)	3.4±0.3	7.7±0.6	<0.001
M/I-value^3^ x 10^−2^ [mg/(kg*min)) / (μU/ml)]	2.6±0.3	5.8±0.5	<0.001

^1^ Plasma insulin concentration at steady state during a euglycemic-hyperinsulinemic clamp.

^2^ M-value = glucose disposal derived from a euglycemic-hyperinsulinemic clamp.

^3^ M/I-value = insulin sensitivity derived from a euglycemic-hyperinsulinemic clamp. Calves with a M/I-value <4.4 were considered insulin resistant. M/I-values_IR_ ranged from 2.1–3.8 and M/I-values_MIS_ ranged from 4.4–8.2; x 10^−2^ [mg/(kg*min)) / (μU/ml)].

^4^ *P*-value was calculated from independent T-test.

### Metabolomic profiling

**Fig 1:**
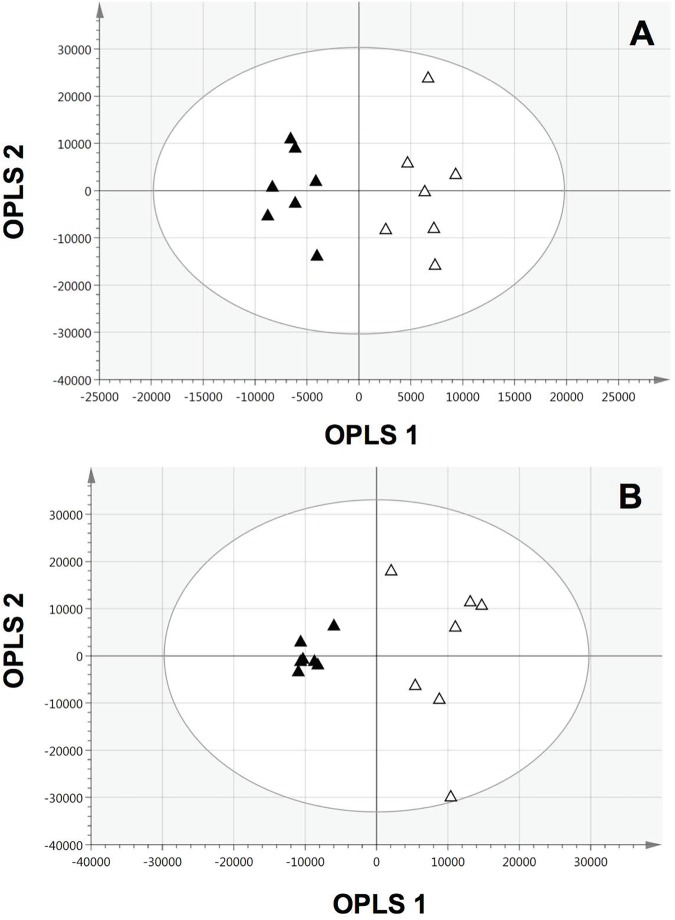
OPLS-DA score plots from plasma metabolic profiles of moderately insulin sensitive and insulin resistant veal calves. The white triangles represent moderately insulin sensitive veal calves (n = 7) and black triangles represent insulin resistant veal calves (n = 7). The models were obtained using C18 LC-MS (A) and HILIC LC-MS (B) blood plasma metabolic profiling. R^2^Y, which is the variation described by the models was 92 and 90% for the C18 and HILIC model, respectively. Q^2^Y, which describes how accurately the models can predict class membership, was 66 and 73% for the C18 and HILIC model, respectively.

Metabolomic profiling of IR *vs*. MIS calves was performed on the fasting plasma samples collected on the day of the clamp study. The models obtained using OPLS-DA are shown in Fig 1. A total of 247 metabolic features were detected using the C18 RP LC-MS. The C18 model clearly distinguished MIS calves from IR calves. The non-orthogonal component of this model explained 92% of the variation (R^2^Y = 0.92). The predictive power of the model, measured by seven-fold cross validation was 66% (Q^2^Y = 0.66). The CV-ANOVA *P*-value was 0.03, indicating that the differences between the two groups were significant. Furthermore, permutation tests (n = 100) were performed to assess the robustness of the model. The validation plots confirmed that the model was valid and unlikely obtained by chance, as the permuted R^2^ and Q^2^ data were lower that original values, and Q^2^ had a negative intercept. A total of 625 metabolic features were detected using the HILIC LC-MS. The HILIC model could also distinguish MIS calves from IR calves. This model explained 90% of the variation (R^2^Y = 0.90). The predictive power was 73% (Q^2^Y = 0.73) and the CV-ANOVA *P*-value was 0.01. Permutation tests (n = 100) confirmed that the model was valid and unlikely obtained by chance. Both the permuted R^2^ and Q^2^ data were lower that original values, and Q^2^ had a negative intercept.

**Fig 2:**
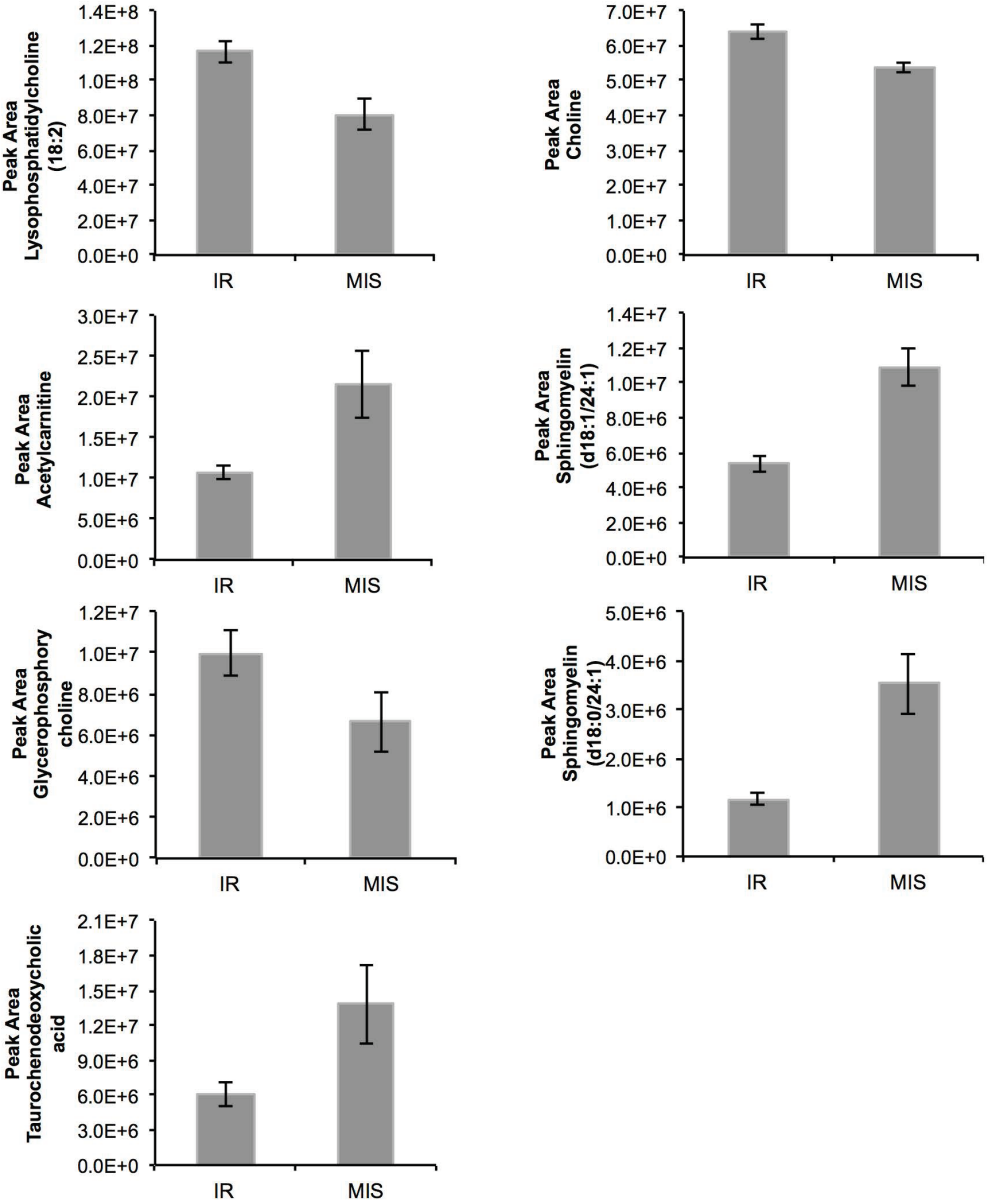
Plasma response of the (putatively) identified biomarkers of insulin resistance in veal calves. The chromatographic peak area is a measure of the blood plasma concentration. MIS = moderately insulin sensitive veal calves (n = 7). IR = insulin resistant veal calves (n = 7). Further details of these two groups are given in Table 1. Error bars represent SEM.

A total of 7 and 20 metabolic features had a variable importance in the projection >2.0 in the C18 and HILIC model, respectively (S1 Table). Of these metabolites, a total of 1 and 11 metabolic features differed (*P* <0.05) between insulin sensitivity groups in the C18 and HILIC model, respectively (Table 2). Seven of these metabolic features decreased in IR calves and five increased. A total of 7 metabolites could be (putatively) identified. The chromatographic response of these metabolites (as a measure of the plasma concentration) is given in Fig 2.

**Table 2:** Marker metabolites of insulin resistance found in OPLS-DA models of HILIC and C18 LC-MS plasma metabolic profiling of veal calves.

m/z	VIP^1^	*P*-value^2^	Metabolites^3^	Chemical class	Fold change (IR/MIS)^6^
520.339	8.27^4^	0.005	Lysophosphatidylcholine (18:2)	Lysophospholipids	1.45
703.574	5.47^4^	0.013		Sphingomyelins	0.70
104.107	4.48^4^	0.002	Choline	Cholines	1.19
204.123	3.65^4^	0.031	Acetylcarnitine	Acyl carnitines	0.50
185.127	3.61^4^	0.005		Sphingomyelins	1.15
813.682	3.57^4^	0.002	Sphingomyelin (d18:1/24:1)	Sphingomyelins	0.49
498.288	2.91^5^	0.017	Taurochenodeoxycholic acid	Bile acids	0.44
811.668	2.47^4^	0.001		Sphingomyelins	0.56
258.110	2.46^4^	0.041	Glycerophosphorylcholine	Glycerophosphorylcholines	1.50
815.698	2.18^4^	0.000	Sphingomyelin (d18:0/24:1)	Sphingomyelins	0.33
787.668	2.10^4^	0.001		Sphingomyelins	0.95
564.330	2.02^4^	0.034			1.34

^1^ Variable importance in the projection (VIP) obtained from OPLS-DA models with a threshold of ≥2.0.

^2^ *P*-value was calculated from independent samples T-test. Threshold was set at *P* ≤0.05.

^3^ (Putative) identification based on human metabolome database and METLIN database search combined with MS/MS fragmentation analysis, and in some cases, comparison with authentic standards (when available in house).

^4^ VIP obtained from the HILIC OPLS-DA model.

^5^ VIP obtained from the C18 OPLS-DA model.

^6^ IR = Insulin resistant calves. MIS = moderately insulin sensitive calves.

### Pathway analysis

Pathway analysis was performed on the 7 putatively identified metabolites using Metaboanalyst to highlight pathways possibly associated with insulin resistance in veal calves. The 7 putatively identified metabolites are involved in 4 pathways; the glycerophospholipid metabolism, sphingolipid metabolism, glycine, serine and threonine metabolism, and primary bile acid biosynthesis (Table 3).

**Table 3:** Metabolic pathway analysis of the putative identified marker metabolites of insulin resistance (Table 1) found in blood plasma of veal calves.

Metabolic pathway^1^	Marker metabolite	Trend^2^
Glycerophospholipid metabolism	Lysophosphatidylcholine (18:2)	↑
	Choline	↑
	Glycerophosphorylcholine	↑
Sphingolipid metabolism	Sphingomyelin (d18:1/24:1)	↓
	Sphingomyelin (d18:0/24:1)	↓
Glycerine, serine and threonine metabolism	Choline	↑
Primary bile acid biosynthesis	Taurochenodeoxycholic acid	↓

^1^ Metabolic pathway analysis was performed using MetaboAnalyst version 2.0.

^2^ ↑ and ↓ indicate that the marker was increased or decreased in insulin resistant claves compared with moderately insulin sensitive calves.

## Discussion

Plasma metabolic profiling techniques have been applied to build models to find biomarkers and pathways that can identify insulin resistant veal calves and distinguish these calves from moderately insulin sensitive calves. To the best of our knowledge, this is the first time that metabolic profiling has been applied on veal calves to study insulin resistance. Satisfactory models (Q^2^Y = 66 and 73% for C18 and HILIC, respectively) were developed, that could clearly identify insulin resistant veal calves, and which could possibly be used in early diagnosis. The predictive powers of these models are slightly lower compared to human metabolic profiling studies (Q^2^Y = 76–93%; [14, 15]). This might be attributed to multiple factors: 1] the small number of calves used in this study 2] possible differences in the metabolic profiling techniques used, and 3] possible differences in degree of the experimental contrasts in insulin sensitivity between human and calf studies. In veal calves, insulin sensitivity decreases substantially within the first weeks of life [5], which leads to smaller contrasts in insulin sensitivity in later life. One possible source of variation that can be excluded from subsequent studies is the use of multiple dietary treatments. Despite the fact that insulin sensitivity was not differentially affected by the dietary treatments (dietary treatments almost balanced out between classification groups), it could be that certain metabolites that are more strongly affected by dietary treatment. Nonetheless, the potential biomarkers found in this study were not affected by dietary treatment (as assessed by independent t-test; *P*-values > 0.05). In subsequent studies, it might be beneficial to restrict feeding to the standard (commercial) lactose MR diet. Another source of variation that can be excluded from subsequent studies is the possible effect of age on insulin sensitivity. In our study, insulin sensitivity was measured within a period of 7 weeks (2 calves per week). The possible effect of age on insulin sensitivity, however, was balanced out between classification groups, as age did not differ between groups. In subsequent studies, insulin sensitivity should be measured at the same time (day). Additionally, it would be interesting to measure insulin sensitivity in time (i.e. with age) in the same set of calves to study the discovered biomarkers and their association with the development of insulin resistance with age.

A M/I cut-off value of 4.4 was used to discriminate between insulin resistant and moderately insulin sensitive calves. This value was based on cut-off values for defining insulin resistance in humans, because cut-off values for calves are not established [6]. A different cut-off value would have perhaps let to discovery of additional/other biomarkers (and pathways). Nevertheless, the cut-off value used in this study let to clear discrimination between insulin sensitivity groups (i.e. a low *vs*. moderate group). Therefore, the discovered biomarkers are related to differences in insulin sensitivity levels.

In our study, not all potential biomarkers could be identified. This is a well-known bottleneck of untargeted MS metabolic profiling techniques [16, 17]. Future studies should also consider including additional identification techniques such as nuclear magnetic resonance spectroscopy.

Interestingly, pathway analysis of the putatively identified potential biomarkers revealed multiple disturbances in the glycerophospholipid and the sphingolipid metabolism. To the best of our knowledge, this is the first time that these pathways (and potential biomarkers) have been associated with insulin resistance in veal calves. This demonstrates the power of metabolic profiling in identifying markers and pathways that may be important in understanding the development of insulin resistance in calves. In dairy cows, both pathways have recently been associated with reduced insulin sensitivity [18]. In humans, these pathways have frequently been associated with insulin resistance and type 2 diabetes [19–23]. A previous study has shown that different sphingolipids associate either positively or negatively with insulin resistance [19]. In human and rodents, sphingomyelins (a type of sphingolipid) patches on β-cells and predicts insulin secretory capacity [24]. Decreased glucose tolerance and insulin secretion have been observed in sphingomyelin synthase 1 knockout mice [25, 26]. Our data also show that not all sphingomyelins are negatively associated with insulin resistance. The mechanisms behind the different associations warrant further study. Glycerophospholipids have also been associated with insulin resistance and type 2 diabetes. In human studies, both positive and negative associations have been found for metabolites related to glycerophospholipid metabolism [27–30]. In our study a positive association was found. Glycerophospholipids are major components of cell membranes. Disturbances in membrane glycerophospholipid metabolism could influence insulin secretion via alteration of the physico-chemical properties of the membrane [20]. However, clear mechanisms behind the associations of metabolites related to glycerophospholipid metabolism have not been identified yet. Future (mechanistic) studies on the development of insulin resistance in calves should apply a targeted lipidomic approach that specifically focuses on metabolites related to the glycerophospholipid and the sphingolipid metabolism.

## Conclusion

Based on plasma metabolic profiling satisfactory models were developed that are capable of distinguishing veal calves differing in insulin sensitivity (i.e. moderate vs. insulin resistant/extremely low insulin sensitive calves). Several metabolic alterations (potential biomarkers) were observed between the moderate and low insulin sensitive calves. These alterations were related to the glycerophospholipid metabolism, sphingolipid metabolism, glycine, serine and threonine metabolism, and primary bile acid biosynthesis. Future studies should be performed to study these pathways and biomarkers in early life (i.e. neonatal calves) and their association with the development of insulin resistance with age in veal calves.

## Supporting information

S1 Table Metabolic features (with VIP ≥2.0) found in OPLS-DA models of HILIC and C18 LC-MS plasma metabolic profiling of moderately insulin sensitive and insulin resistant veal calves. (DOCX)
