# Supplementary material for: The use of metabolic profiling to identify insulin resistance in veal calves
Source: PLoS One. 2017 Jun 15;12(6):e0179612. doi: 10.1371/journal.pone.0179612 (PMC5472311; doi:10.1371/journal.pone.0179612)
Supplement: S1 Table — (DOCX) [file pone.0179612.s001.docx]

| **Metabolic feature** | **m/z** | **Mode^1^** | **VIP^2^** |
| --- | --- | --- | --- |
| 1 | 520.339 | HILIC | 8.27 |
| 2 | 703.574 | HILIC | 5.47 |
| 3 | 104.107 | HILIC | 4.48 |
| 4 | 524.370 | HILIC | 3.70 |
| 5 | 204.123 | HILIC | 3.65 |
| 6 | 185.127 | HILIC | 3.61 |
| 7 | 496.343 | HILIC | 3.59 |
| 8 | 524.374 | HILIC | 3.59 |
| 9 | 813.682 | HILIC | 3.57 |
| 10 | 498.289 | HILIC | 3.47 |
| 11 | 464.282 | HILIC | 2.97 |
| 12 | 522.355 | HILIC | 2.57 |
| 13 | 414.302 | HILIC | 2.53 |
| 14 | 811.668 | HILIC | 2.47 |
| 15 | 258.110 | HILIC | 2.46 |
| 16 | 432.311 | HILIC | 2.34 |
| 17 | 815.698 | HILIC | 2.18 |
| 18 | 116.071 | HILIC | 2.14 |
| 19 | 787.668 | HILIC | 2.10 |
| 20 | 564.330 | HILIC | 2.02 |
| 1 | 520.340 | C18 | 6.79 |
| 2 | 496.339 | C18 | 4.59 |
| 3 | 524.370 | C18 | 4.53 |
| 4 | 789.619 | C18 | 3.32 |
| 5 | 522.355 | C18 | 3.14 |
| 6 | 498.288 | C18 | 2.91 |
| 7 | 464.283 | C18 | 2.00 |

^1^ Chromatographic mode used. HILIC = Hydrophilic interaction chromatography and C18 = reversed phase C18 chromatography.

^2^ VIP = Variable importance in the projection, obtained from the OPLS-DA models
